# PSMA Expression Predicts Early Biochemical Response in Patients with Metastatic Castration-Resistant Prostate Cancer under ^177^Lu-PSMA-617 Radioligand Therapy

**DOI:** 10.3390/cancers13122938

**Published:** 2021-06-11

**Authors:** Liam Widjaja, Rudolf A. Werner, Tobias L. Ross, Frank M. Bengel, Thorsten Derlin

**Affiliations:** Department of Nuclear Medicine, Hannover Medical School, 30625 Hannover, Germany; liam.widjaja@stud.mh-hannover.de (L.W.); ross.tobias@mh-hannover.de (T.L.R.); bengel.frank@mh-hannover.de (F.M.B.); derlin.thorsten@mh-hannover.de (T.D.)

**Keywords:** prostate carcinoma, PSMA, radioligand therapy, PET/CT, biochemical response, mCRPC

## Abstract

**Simple Summary:**

Prostate-specific membrane antigen (PSMA) is a promising target for both imaging and radioligand therapies (RLT) for men with prostate cancer. However, not all patients respond to RLT and some even progress early in the treatment course. We aimed to identify parameters to forecast which patients will achieve therapy response prior to commencing RLT. Therefore, we tested whether the tumor volume, the level of PSMA expression or a combination of both in metastases derived from PSMA-targeted molecular imaging prior to RLT can inform the treating physician whether a patient will respond to RLT. Compared to tumor volume, the level of PSMA-expression can better identify patients responding to RLT early in the treatment course.

**Abstract:**

^177^Lu-Prostate-specific membrane antigen (PSMA)-radioligand therapy (RLT) is a promising treatment option in patients with metastatic castration-resistant prostate cancer (mCRPC). We aimed to determine the predictive value of pretherapeutic PSMA-ligand positron emission tomography (PET) and established clinical parameters for early biochemical response after two cycles of RLT. In total, 71 mCRPC patients who had undergone PET/computed tomography (CT) with ^68^Ga-PSMA-11 prior to two cycles of ^177^Lu-PSMA-617 RLT were included. Malignant lesions on pretherapeutic PET/CTs were manually segmented and average maximum PSMA expression (maximum standardized uptake values, SUV_max_), whole-body PSMA-tumor volume (TV), and whole-body total lesion (TL)-PSMA were calculated. We then tested the predictive performance of these parameters for early biochemical response (defined as prostate-sepcific antigen (PSA) decrease of ≥50% according to PCWG2) after two cycles of RLT, relative to established clinical parameters. Early PSA response was observed in 34/71 patients. PSA change after two cycles of RLT correlated with pretherapeutic SUV_max_ (r = −0.49; *p* < 0.001), but not with PSMA-TV (r = 0.02; *p* = 0.89) or TL-PSMA (r = −0.15; *p* = 0.22). A cut-off of 19.8 for SUV_max_ and 75.5 years for age was defined by receiver operating characteristics and revealed a significant outcome difference for early biochemical response between patients with adversely low vs. high PSMA expression and low vs. high age (*p* < 0.001). Multivariate analysis identified SUV_max_ (HR, 7.94, *p* = 0.001) and age (HR, 8.05, *p* = 0.002) as independent predictors for PSA response early in the treatment course. Thus, high age and high PSMA expression in patients scheduled for RLT identify patients with early biochemical response. This study provides a rationale for further prospective studies exploring PET-guided treatment intensification in selected patients.

## 1. Introduction

Recent years have witnessed an expanded use of prostate-specific membrane antigen (PSMA)-targeted imaging and therapy for men afflicted with metastatic castration-resistant prostate cancer (mCRPC) [1,2,3,4,5,6]. For instance, PSMA-targeted positron emission tomography (PET)/computed tomography (CT) has gained increasing acceptance in the diagnosis of prostate cancer (PC) due to its superior accuracy in identifying metastases compared to both CT and magnetic resonance imaging (MRI) [7,8]. Moreover PSMA-targeted radioligand therapy (RLT) with ^177^Lu-PSMA demonstrated great potential in patients with mCRPC in several retrospective and prospective studies [1,2,9,10].

These preliminary beneficial results were further corroborated by a prospective phase 2 study performed by Hofman et al., which reported on both preferable response rates and less adverse events in mCRPC patients undergoing ^177^Lu-PSMA RLT compared to the established chemotherapeutic cabazitaxel [11]. In addition, the ongoing prospective VISION phase-3 trial further investigates the clinical value of PSMA RLT and hence may further boost the use of RLT in mCRPC patients [12]. Most studies demonstrated PSA response in about half of the enrolled subjects, whereas 30% of the patients still suffered from progressive disease (PSA increase of >25%) [13]. Therefore, predictors for identifying such high-risk individuals are intensively sought, and multiple studies have already investigated the use of PSMA-ligand PET/CT for late outcome, e.g., in terms of overall survival (OS). Seifert et al. demonstrated a significant association between elevated PSMA expression (defined as average maximum standardized uptake value [SUV_max_]) and prolonged survival [14], whereas increasing tumor volume on PSMA-ligand PET (PSMA- tumor volume [TV]) was associated with worse outcome [15]. Pooling over 260 mCRPC patients from six different centers, however, contrary findings on long-term PSA-based progression-free survival were described, indicating that higher PSMA-avid tumor burden was associated with more favorable OS [16]. Such contrary findings on long-term outcome may underpin the urgent need to identify predictors early in the treatment course, preferably in a pretherapeutic setting. Such short-term prognosticators in mCRPC patients under RLT would be helpful to alter therapeutic management or guide the treating physician towards other therapeutic regimens early in the disease course. Therefore, in the present study, we aimed to investigate the predictive performance of pretherapeutic ^68^Ga-PSMA-11 PET/CT-derived parameters relative to established clinical parameters to predict early biochemical response after two cycles of ^177^Lu-PSMA RLT.

## 2. Materials and Methods

### 2.1. Patient Population

In total, 71 mCRPC patients (72.1 ± 7.21 years; range 52–85 years) who underwent a ^68^Ga-PSMA-11 PET/CT and subsequent ^177^Lu-PSMA-617 RLT between June 2016 and August 2019 were included in this retrospective, single-center study (Table 1). All patients suffered from progressive disease after previous therapies including androgen deprivation therapy, and second-line regimens such as abiraterone acetate, enzalutamide or taxane-based chemotherapy or were unfit for chemotherapy [17]. All interventions were performed as part of clinical routine care. ^177^Lu-PSMA-617 was administered in accordance with the Declaration of Helsinki (“unproven interventions in clinical practice”) and the German Medicinal Products Act, AMG §13.2b.

The institutional review board approved the study (No. 9182_BO_S_2020, Hannover Medical School). All patients provided written informed consent for all therapeutic and diagnostic procedures and for the retrospective data analysis. Parts of this cohort have also been investigated in [17].

### 2.2. Imaging Procedure

All studies were performed using a hybrid PET/CT system (Biograph mCT 128 Flow; Siemens Healthineers; Knoxville, TN, USA), with an extended field-of-view for the PET and a 128-slice spiral CT, as previously described [18]; 107 ± 23 MBq of ^68^Ga-PSMA-11 was administered. First, a low-dose non-enhanced helical CT (120 kV, mA modulated, pitch of 1.2, reconstructed axial slice thickness of 5.0 mm) for attenuation correction was performed, followed by a whole-body PET using continuous bed motion at a speed of 0.9 mm/s for chest and abdomen and 2.1 mm/s for legs at 1 h post injection. All studies were reconstructed using Ultra HD (iterative algorithm with time-of-flight and point-spread function information including two iterations, 21 subsets; matrix, 200; zoom, 1.0; gaussian filter, 5.0). No intravenous contrast material was injected.

### 2.3. Image Interpretation and Calculation of Quantitative PET Parameters

All PET/CT images were analyzed using a commercial software package (syngo.via; V50B; Siemens Healthcare) [18]. Malignant lesions on all scans were identified by a single reader (LW) and confirmed by an experienced reader (RAW). For every lesion, an isocontour volume of interest including all voxels above 45% of the maximum was created using a 3-dimensional segmentation method allowing for a semi-automatic volumetric assessment, as described previously [18]. Such an approach enabled calculation of the whole-body PSMA-TV and whole-body total lesion (TL)-PSMA, defined as PSMA-TV x mean standardized uptake value (SUV_mean_) [18]. SUV_mean_ and SUV_max_ were both derived from volume of interests. Average SUV_max_ of all lesions per patient was then calculated, as suggested for long-term outcome analysis of mCRPC patients under RLT [14]. Moreover, localization of every lesion (skeleton, lymph nodes, liver, prostate or soft tissue) was noted. This approach also allowed for calculating the average SUV_max_, whole-body PSMA-TV, and TL-PSMA separately for every investigated organ compartment.

### 2.4. Lu-177-PSMA-617 RLT

A GMP-compliant synthesis of ^177^Lu-PSMA-617 was conducted as described previously [17]. Patients received 6 to 7.4 GBq of ^177^Lu-PSMA-617 every 6–8 weeks (total dose of 13.31 ± 2.18 per patient). Treatment was conducted in adherence with the national consensus recommendation [19], with treatment cycles repeated every 6–8 weeks.

### 2.5. Assessment of Early PSA Response

Response to treatment was assessed in Cycle 3 Day 1 using PSA levels according to Prostate Cancer Clinical Trials Working Group criteria (PCWG2) [20]. In patients discontinuing RLT before the 3rd cycle, last available PSA was used for response assessment. The PCWG2 criteria define response as a 50% or greater decrease in prostate-specific antigen (PSA) levels [20], which was also used in the present analysis. PSA response rate and the percentage PSA decline after two cycles (i.e., in Cycle 3 Day 1) were recorded for each patient, which enabled an early biochemical response assessment of RLT [17]. In patients who discontinued PSMA-directed treatment, the PSA level on the date of therapy cessation was used for further analyses [17].

In addition, a standard blood panel collected prior to RLT was included in the analysis and comprised red blood cells (RBC, in 10^6^/μL), white blood cells (WBC, in 10^3^/μL), platelets (in 10^3^/μL), aspartate aminotransferase (AST, in U/L), alanine aminotransferase (ALT, in U/L), lactate dehydrogenase (LDH, in U/L), and alkaline phosphatase (AP, in U/L).

### 2.6. Statistical Analysis

Statistical analysis was done with GraphPad Prism 9 (GraphPad Software; San Diego, CA, USA) and SPSS Statistics 27 Inc. (IBM, Chicago, IL, USA). Waterfall plots were used for graphical illustration of PSA response. Heat maps were used for graphical illustration of PSMA expression and PSMA-TV among patients. The Kruskal–Wallis test was used to compare two independent groups, and simple linear regression was used to estimate the relationship between two parameters. We used one-way analysis of variance to determine whether PSMA-TV, TL-PSMA or PSMA expression differed between different Gleason Scores or tumor localizations. Fisher’s exact test was performed to assess the relationship between PSA response and PSMA expression, PSMA-TV, TL-PSMA or other parameters. Cut-offs for the prediction of PSA response were determined by receiver operating characteristics (ROC) analysis, using the Youden Index for maximization of specificity and sensitivity. Univariate Kaplan–Meier analysis was then performed using cut-offs established by previously conducted ROC, and the nonparametric log-rank test was used to determine significant outcome differences between subgroups. Finally, multivariate logistic regression analysis was used to identify independent predictors of early biochemical response [21]. A *p*-value of <0.05 was considered to be statistically significant.

## 3. Results

Patient characteristics stratified into responders and non-responders are displayed in Table 1. Responders were significantly less frequently pretreated with chemotherapy (*p* = 0.045) and less frequently had hepatic metastases (*p* = 0.009). In addition, the time period between initial diagnosis of prostate cancer and 1st RLT was significantly longer in patients responding to RLT (*p* = 0.004). In line with this finding, responders were also significantly older (*p* < 0.001).

### 3.1. SUV_max_ and PSMA-TV Are Independent Parameters for Baseline ^68^Ga-PSMA-11 PET/CT

Analyzing the pretherapeutic ^68^Ga-PSMA-11 PET/CTs, 5694 malignant classified lesions were detected. Per patient, average SUV_max_ was 20.89 ± 18.11, the average whole-body TL-PSMA was 2878.37 ± 2792.28 cm^3^, and the average PSMA-TV was 215.82 ± 219.43 cm^3^ with the dominant tumor burden being located in the skeleton (153.6 ± 195 cm^3^) followed by the liver (29.07 ± 133.4 cm^3^), lymph nodes (25.88 ± 38.28 cm^3^), other (5.78 ± 20.13 cm^3^), and prostate (1.04 ± 2.57 cm^3^, *p* < 0.001). There was no statistically significant correlation between SUV_max_ and PSMA-TV derived from baseline PET (r = −0.16, *p* = 0.20), supporting the notion that both parameters should be investigated separately for further outcome analysis. In contrast, both SUV_max_ (r = 0.26, *p* = 0.04) and PSMA-TV (r = 0.79, *p* < 0.001) correlated with TL-PSMA. SUV_max_ (*p* = 0.18), PSMA-TV (*p* = 0.20), and TL-PSMA (*p* = 0.08) were not associated with the Gleason score. In addition, PSMA-expression (SUV_max_) did not differ between the different tumor localizations (skeleton, 17.11 ± 9.9; lymph nodes, 21.49 ± 20.47; liver, 12.91 ± 5.36; prostate, 15.75 ± 10.74 and other, 18.64 ± 18.1; *p* = 0.34).

### 3.2. SUV_max_, but Not PSMA-TV or TL-PSMA, Is Associated with PSA Change under RLT

**Figure 1:**
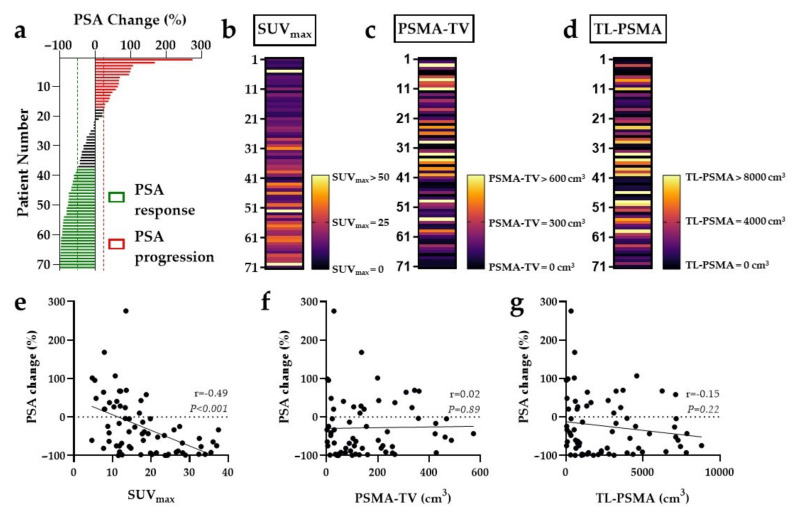
Change of prostate-specific antigen (PSA) values relative to maximum standardized uptake value (SUV_max_), prostate-specific membrane antigen-based tumor volume (PSMA-TV) and total lesion (TL)-PSMA. (**a**) Waterfall plot of PSA change after two cycles of ^177^Lu-PSMA-617 radioligand therapy presented in order of increasing PSA response. Interindividual heterogeneity in SUV_max_ levels (**b**), PSMA-TV (**c**), and TL-PSMA (**d**) derived from baseline ^68^Ga-PSMA-11 PET/CT prior to therapy (black indicates low SUV, PSMA-TV, and TL-PSMA, yellow indicates high SUV, PSMA-TV, and TL-PSMA). In contrast to both PSMA-TV and TL-PSMA, high SUV_max_ is associated with PSA response, as can be seen in the waterfall plot of PSA change. Consequently, the change in PSA correlated significantly with SUV_max_ (**e**), but not with PSMA-TV (**f**) and TL-PSMA (**g**).

A median PSA change of −43.4% (IQR, −84.7% to 21.6%) after two cycles of RLT was recorded, and 34 (47.9%) patients demonstrated early biochemical response according to PCWG2 (Figure 1a). A higher SUV_max_ (r = −0.49, *p* < 0.001, Figure 1b,e) was associated with PSA decline. In contrast, both PSMA-TV (r = 0.02, *p* = 0.89; Figure 1c,f) and TL-PSMA (r = −0.15, *p* = 0.22; Figure 1d,g) did not correlate with the PSA change after two cycles of RLT.

### 3.3. SUV_max_ and Age Are Independent Predictors of Early PSA Response

We performed a ROC analysis to evaluate whether clinical or PET-derived parameters are accurate in identifying patients with and without PSA response and to define optimal thresholds for predicting PSA response after two cycles of RLT. SUV_max_ demonstrated high significance in ROC analysis with an area under the curve (AUC) of 0.73 (*p* < 0.001, best threshold ≥19.8). In contrast, PSMA-TV (AUC = 0.55, *p* = 0.44, best threshold ≤130 cm^3^), and TL-PSMA (AUC = 0.58, *p* = 0.22, best threshold ≥588 cm^3^) failed to reach significance. ROC-based most suitable thresholds were also identified for the clinical parameters age (AUC = 0.74, *p* < 0.001, best threshold ≥75.5 years), the time period between initial diagnosis and 1st RLT (AUC = 0.69, *p* = 0.003, best threshold ≥10.5 years), RBCs (AUC = 0.58, *p* = 0.22, best threshold ≥3.9 × 10^6^/μL), LDH (AUC = 0.57, *p* = 0.35, best threshold ≤308 U/L), PSA (AUC = 0.55, *p* = 0.46, best threshold ≤70 μg/L), WBCs (AUC = 0.55, *p* = 0.47, best threshold ≥7.3 × 10^3^/μL), AP (AUC = 0.55, *p* = 0.5, best threshold ≤153.5 U/L), AST (AUC = 0.54, *p* = 0.54, best threshold ≤26.5 U/L), ALT (AUC = 0.51, *p* = 0.85, best threshold ≤15.5 U/L), and platelets (AUC = 0.5, *p* = 0.94, best threshold ≥230 × 10^3^/μL).

In univariate analysis (Table 2), SUV_max_ (Odds Ratio [OR], 8.35, 95%CI, 2.74–25.45, *p* < 0.001), age (OR, 8.11, 95%CI, 2.54–25.87, *p* < 0.001), and the time period between initial diagnosis and 1st RLT (OR, 7.33, 95%CI, 2.13–25.27, *p* = 0.001) demonstrated predictive values for early biochemical response after two cycles of RLT, as well as by TL-PSMA (OR, 5.6, 95%CI, 1.43–21.89, *p* = 0.01). By contrast, hepatic metastases were associated with subsequent worse outcome (OR, 0.15, 95%CI, 0.03–0.73, *p* = 0.01). None of the other parameters demonstrated predictive values for PSA response.

**Figure 2:**
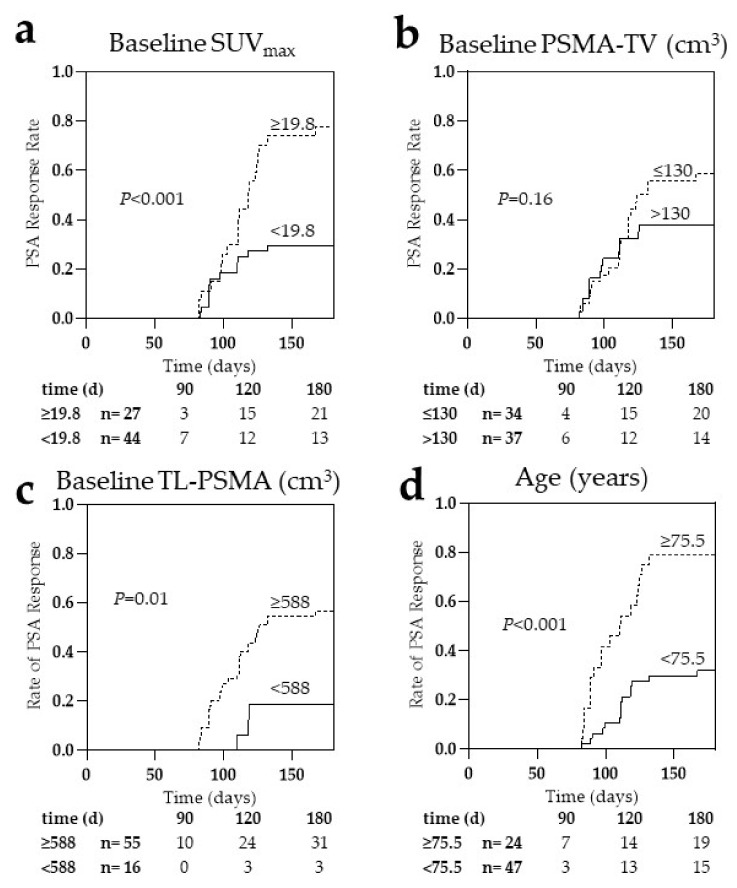
Kaplan–Meier curves for early prostate-specific antigen (PSA) response, using maximum standardized uptake value (SUV_max_) (**a**), prostate-specific membrane antigen-based tumor volume (PSMA-TV) (**b**), total lesion (TL)-PSMA (**c**), and age (**d**) prior to radioligand therapy. Higher SUV_max_, age, and TL-PSMA were significantly associated with the PSA response, whereas PSMA-TV was not significantly associated. d = days.

In univariate Kaplan–Meier analysis, an SUV_max_ higher than 19.8 and an age older than the 75.5-year threshold were strongly associated with early PSA response (*p* < 0.001; Figure 2a,d), followed by TL-PSMA (*p* = 0.01; Figure 2c), while PSMA-TV did not reach significance (*p* = 0.16; Figure 2b).

**Figure 3:**
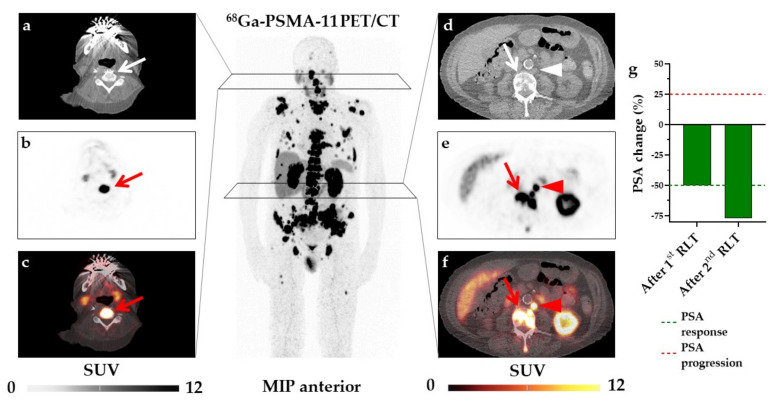
Case example of a responder. Representative metastases in the 4th cervical vertebra (arrow, SUV_max_ = 62.85, PSMA-TV = 2.9 cm^3^) on transaxial CT (**a**), PET (**b**) and fused ^68^Ga-PSMA-11 PET/CT (**c**), the 3rd lumbar vertebra (arrow, SUV_max_ = 91.1, PSMA-TV = 5.56 cm^3^, **d**–**f**) and a paraaortic lymph node (arrowhead, SUV_max_ = 59.95, PSMA-TV = 0.33 cm^3^; **d**–**f**). Maximum intensityprojection (MIP) image for orientation (middle panel). In this patient, the overall PSMA-TV was 256.86 cm^3^, i.e., above the ROC-derived threshold of 130 cm^3^ (indicative for early biochemical failure). Overall SUV_max_ (32.97) was also above the ROC-based threshold, which is associated with early biochemical response to radioligand therapy (RLT). PSA decreased by 77% (indicating PSA response according to PCWG2) after two cycles of ^177^Lu-PSMA RLT (**g**), thereby demonstrating that SUV_max_ and age (79 years, also indicative for response) had a better predictive performance relative to PSMA-TV.

**Figure 4:**
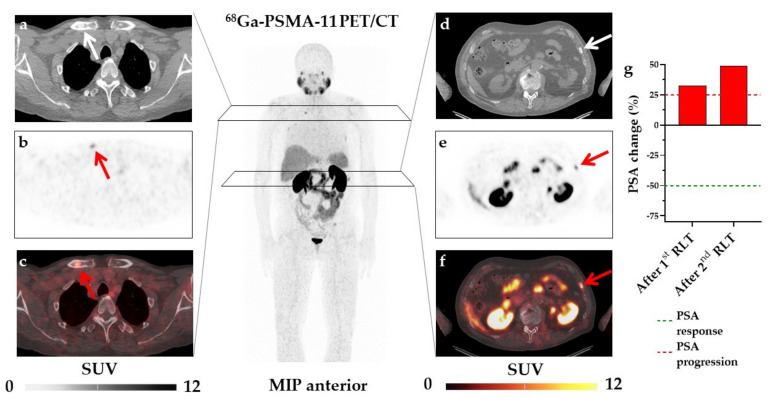
Case example of a non-responder. Representative osseous metastases in the right clavicle (arrow, SUVmax = 6.11, PSMA-TV = 0.83 cm^3^) on transaxial CT (**a**), PET (**b**) and fused ^68^Ga-PSMA-11 PET/CT (**c**) and in the left 9th rib (arrow, SUV_max_ = 7.7, PSMA-TV = 1 cm^3^, **d**–**f**). Maximum intensity projection (MIP) image for orientation (middle panel). In this patient, the overall PSMA-TV was 18.01 cm^3^, i.e., below the ROC-derived threshold of 130 cm^3^ (indicative for early biochemical response). Overall SUV_max_ (5.77) was also below the ROC-based threshold, which is associated with early biochemical failure to radioligand therapy (RLT). PSA increased by 49% (indicating no PSA response according to PCWG2) after two cycles of ^177^Lu-PSMA RLT (**g**), consistent with non-response, thereby demonstrating that SUV_max_ and age (72 years, also indicative of biochemical failure) had a better predictive performance relative to PSMA-TV.

Finally, we performed a multivariate logistic regression analysis including those parameters that demonstrated significance in univariate analysis. However, due to covariance of SUV_max_ and TL-PSMA (r = 0.26, *p* = 0.04), we decided to include only SUV_max_ because of its superior performance in both univariate and Kaplan–Meier analyses. Similarly, we decided to exclude the time period between initial diagnosis and 1st RLT due to covariance with age (r = 0.5, *p* < 0.001) and its inferior performance in ROC- and univariate analysis. Accordingly, we used baseline SUV_max_, age, and hepatic metastases for multivariate analysis. Both age (HR, 8.05, 95%CI, 2.15–30.09, *p* = 0.002) and SUV_max_ (HR, 7.94, 95%CI, 2.25–28.05, *p* = 0.001) emerged as independent predictors of early biochemical response, whereas hepatic metastases did not reach significance (Table 3). Case examples are shown in Figure 3 and Figure 4.

## 4. Discussion

In the present study, both patient age and PSMA expression from pretherapeutic PET/CT emerged as independent predictors for early biochemical response after two cycles of ^177^Lu-PSMA-RLT. These parameters outperformed both the PET-based parameters PSMA-TV and TL-PSMA. Thus, enrolling a substantial and homogenously treated cohort of men afflicted with mCRPC, we could demonstrate the predictive value of PSMA expression and age early in the treatment course. Hence, by using the herein presented cut-offs for the latter parameters, patients prone to early treatment failure could be identified and carefully monitored for early treatment modification.

Hofman et al. recently revealed the favorable safety and efficacy profile of ^177^Lu-PSMA-RLT compared to the standard chemotherapy with cabazitaxel [11]. Further building on these promising results, the VISION phase-3 study currently investigates the clinical value of RLT compared to standard therapies in end-stage disease [12]. In light of the anticipated increased use of ^177^Lu-PSMA theranostics [22], outcome predictors are intensively sought. For instance, Rathke et al. recently demonstrated that baseline PSA had no prognostic value for response prediction in mCRPC patients with RLT, while elevated LDH was associated with an increased frequency of progression [23]. In another study, AP emerged as an independent predictor of changes in PSA in patients with bone metastases under ^177^Lu-PSMA RLT [24]. Interestingly, in our cohort, none of these parameters reached significance in uni- or multivariate analysis. Tumor uptake in salivary glands on posttherapeutic imaging, however, seemed to be highly useful for prediction of treatment response [17,23]. Although easy to implement in clinical routine, posttherapeutic whole-body scintigraphy is not available prior to onset of RLT.

Nonetheless, parameters for reliable outcome prediction should be available before commencing ^177^Lu-PSMA targeted therapy. In this regard, recent studies primarily focused on the predictive performance of multiple clinical and PET-based parameters for long-term outcome, including progression-free and overall survival after numerous cycles of RLT. Seifert et al. were among the first to report on the predictive value of ^68^Ga-PSMA-ligand PET in patients scheduled for RLT and concluded that low PSMA expression reflected by SUV_max_ might be a negative prognosticator for shorter OS [14]. Further corroborating these results, we could also report on the predictive value of average SUV_max_ by investigating an earlier endpoint in the treatment course (cycle 3 day 1). Of note, early prognosticators of treatment response are also of relevance for long-term survival, as changes in PSA have been linked to respective disease-specific death. Gadot and coworkers elegantly demonstrated that a PSA decrease of ≥50% correlated significantly with OS [25], which was further confirmed by other research groups [26,27]. Moreover, a recent meta-analysis also reported a pooled HR of 0.29 for OS for any PSA decline under RLT (*p* < 0.00001) [22]. Taken together, building on the findings of Seifert et al. reporting on the predictive capability of SUV_max_ for long-term survival, we herein demonstrate that this parameter also emerged as the sole independent predictor for early biochemical response. This underpins the clinical relevance of increased PSMA expression for pretherapeutic PSMA-PET/CT for both early and late outcome in patients under RLT. Therefore, although future prospective studies are definitely warranted, low SUV_max_ on PSMA-directed PET could prevent the interpreting molecular imaging expert from recommending RLT. Nonetheless, difference in scanners and treatment protocols from center to center have to be considered, but the herein presented threshold of 19.8 (for early response) along with the optimal cutoff for OS of 14.3 (as shown by Seifert and coworkers [14]) provides evidence that patients below the latter cutoff have an increased likelihood of not responding to treatment.

Beyond SUV_max_, we did not find a predictive value of PSMA-TV early in the treatment course, lacking significance in both uni- and multivariate analyses. Moreover, TL-PSMA only demonstrated an inferior predictive value in univariate analysis when compared to SUV_max_. Thus, regarding the covariance of TL-PSMA and SUV_max_, we refrained from further including TL-PSMA in multivariate analysis, in particular as SUV_max_ had already demonstrated an increased OR of 8.35 relative to TL-PSMA (5.6). In line with our findings, a recent retrospective study enrolling ten patients reported no association between the deltas of PSA and PSMA-TV after two cycles of RLT, which was in contrast to a good correlation of PSA and PSMA-TV before commencing RLT [28]. Despite its reduced predictive capability for early biochemical response, a recently published bicentric analysis also evaluated patients treated with ^177^Lu-PSMA-617 and reported that PSMA-TV was a significant prognosticator for OS [15]. However, it has to be questioned whether patients with elevated PSMA-TV had poorer outcome due to missing response to treatment or because of their reduced general condition and higher tumor burden. Moreover, further studies are also needed to determine whether PSMA-TV of a specific organ compartment such as the skeleton causes less favorable long- or short-term outcome. Nonetheless, if patients are going to be risk-stratified for early biochemical response, results of the present study suggest that average PSMA expression may provide more useful guidance whether RLT should be considered. PSMA tumor lesion quotient (TLQ, defined as TV/SUV_mean_), however, has also been advocated to serve as an independent prognostic factor for OS in patients treated with RLT [15]. Therefore, weighting between SUV and TV, future studies may also investigate such sophisticated volumetric quantification approaches for outcome prediction [15]. A dual-radiotracer approach using PSMA-PET and 2-deoxy-2-[F-18]fluoro-D-glucose has also been performed [2,29], and intertumoral heterogeneity in PC assessed by both radiotracers may be of clinical significance for OS in a manner similar to established dual-tracer imaging protocols for neuroendocrine tumors [30,31]. Therefore, future studies may also consider findings on PSMA-PET with simultaneous increased glucose consumption, and thus, the predictive potential of FDG(+)/PSMA(-) lesions for early biochemical response could also be tested.

Of note, besides PSMA-expression for pretherapeutic PET/CT, we identified older age as an independent predictor of PSA response (HR, 8.05, *p =* 0.002). ^177^Lu-PSMA-RLT is established as a treatment option for patients with mCRPC [32]. Thus, all patients included in our study already progressed under several pretreatments, e.g., radical prostatectomy, chemotherapy, and novel hormonal agents. Interestingly, the time period between initial diagnosis of PC and 1st RLT of those patients not responding to RLT was significantly shorter (6.62 ± 3.79 years) when compared to those responding (9.82 ± 5.19 years, *p* = 0.004). Thus, it could be speculated that non-responders suffered from more aggressive tumor phenotypes with subsequent worse prognosis. Future studies should also investigate if younger mCRPC patients suffer from certain more aggressive genomic patterns along with their potential impact on the efficacy of PSMA-targeted RLT [33]. Moreover, in a study investigating >1000 patients, an increased Gleason Score was also linked to the occurrence of new metastases [34]. In the present study, however, the Gleason Score (at time of first diagnosis) was not associated with any PET parameter and did not correlate with early biochemical response, which is in line with the results of Gadot et al. also not reporting associations between PSA decline and the Gleason Score [25].

Some limitations have to be considered: First, the number of patients enrolled in this single-center, retrospective study is limited, and our findings should be re-evaluated in substantial larger cohorts, preferably in a prospective setting. Moreover, in the present study, response was assessed only using the PSA change after two cycles of ^177^Lu-PSMA-RLT. Although easily to obtain in clinical routine and demonstrating a high predictive value for later outcome [13], Han et al. recently revealed significant discordance between PSA and follow-up PSMA-PET/CTs for response assessment pooling 10 different studies [35]. Nonetheless future studies should also include follow-up PSMA-PET/CTs for response assessment (including early biochemical response and OS) to further define the value of changes in PET parameters, such as delta values of PSMA-TV, TL-PSMA or occurrence of new metastases for subsequent outcome. Besides the properties of the specific radiopharmaceutical, SUVs are also influenced by a variety of technical factors including image reconstruction and system resolution [36,37]. In addition, future studies may also test the predictive value of standardized reporting systems for outcome in the setting of mCRPC patients scheduled for RLT [38,39,40]. Furthermore, results of published and ongoing prospective trials using PSMA-directed treatment in end-stage disease may set the scene for a more widespread adoption of this promising treatment earlier in the therapeutic algorithm of PC patients [37]. Therefore, the concept of identifying patients prone to early RLT response will become more relevant, in particular at a disease stage with a broader armamentarium of available treatment options, e.g., in nonmetastatic CRPC. The present study outline may then serve as template for identifying patients prone to treatment failure in such clinical scenarios.

## 5. Conclusions

Patient age and SUV_max_ in pretherapeutic ^68^Ga-PSMA-11 PET/CT demonstrated an independent predictive value for the PSA response after two cycles of RLT. As such, older age and high PSMA expression may guide the interpreting molecular imaging specialist towards recommending RLT, whereas younger age or lower SUV_max_ could alter therapeutic management towards other therapies. Moreover, consistent with the present study investigating early response, previous reports have already reported on the value of PSMA-PET-based SUV_max_ as an independent prognosticator for long-term outcome, which underpins the clinical importance of this parameter for both early and late response prediction. Of note, older age at first RLT was associated with subsequent favorable outcomes.

## Figures and Tables

**Table 1:** Patient characteristics.

Variable	Responders (*n* = 34)	Non-Responders (*n* = 37)	*p*-Value
Age (years, mean ± SD)	75.24 ± 6.6	69.22 ± 6.58	<0.001 *
Time period between initial diagnosis and 1st RLT (years, mean ± SD)	9.82 ± 5.19	6.62 ± 3.79	0.004
Gleason score	8 (7–9)	8 (7–9)	0.986
Previous treatments (%)			
Radical prostatectomy	74	54	0.09
Primary radiation therapy	9	8	0.915
Salvage radiation therapy	59	62	0.778
Androgen deprivation therapy	100	100	1
Novel hormonal agents ^#^	79	92	0.142
Previous chemotherapy	74	92	0.045 *
Median standard laboratory values at baseline (IQR in parentheses)			
RBC (×10^6^/μL)	4.1 (3.8–4.4)	3.9 (3.4–4.3)	0.114
WBC (×10^3^/μL)	4.9 (3.4–6.7)	4.5 (3.5–5.7)	0.789
Platelets (×10^3^/μL)	224 (193–268)	220 (196–274)	0.797
AST (U/L)	26 (23–35)	28 (22–53)	0.244
ALT (U/L)	17 (12–22)	15 (12–22)	0.604
LDH (U/L)	264 (233–326)	307 (221–472)	0.181
AP (U/L)	117 (74–223)	160 (74–337)	0.346
PSA (μg/L)	134 (35–382)	239 (48–483)	0.766
Site of tumor lesions (%)			
Osseous	79	84	0.64
Lymph nodes	76	84	0.446
Hepatic	6	30	0.009 *
Prostate bed	18	30	0.236
Other	29	32	0.787

* reached significance; ^#^ including enzalutamide or abiraterone acetate; Gleason score, red blood cells (RBC), white blood cells (WBC), platelets, aspartate transaminase (AST), alanine transaminase (ALT), lactate dehydrogenase (LDH), alkaline phosphatase (AP), and prostate-specific antigen (PSA) are presented as the median with the interquartile range in parentheses.; prostate-specific membrane antigen (PSMA); radioligand therapy (RLT).

**Table 2:** Univariate predictors for early biochemical response.

Variable	Odds Ratio	95% CI	*p*-Value
*Clinical parameters at baseline*			
RBC (×10^6^/μL)	2.53	0.95 to 6.75	0.09
WBC (×10^3^/μL)	2.34	0.9 to 6.14	0.1
Platelets (×10^3^/μL)	1.29	0.5 to 3.36	0.63
AST (U/L)	0.63	0.24 to 1.61	0.35
ALT (U/L)	0.82	0.31 to 2.22	0.8
LDH (U/L)	0.61	0.22 to 1.67	0.45
AP (U/L)	0.74	0.29 to 1.91	0.63
PSA (μg/L)	0.47	0.17 to 1.27	0.15
Age (years)	8.11	2.54 to 25.87	<0.001 *
Time period between initial diagnosis and 1st RLT (years)	7.33	2.13 to 25.27	0.001 *
*Previous chemotherapy*	0.25	0.06 to 1	0.06
*PET-derived parameters at baseline*			
Hepatic metastases	0.15	0.03 to 0.73	0.01 *
PSMA-TV (cm^3^)	0.43	0.16 to 1.11	0.1
TL-PSMA (cm^3^)	5.6	1.43 to 21.89	0.01 *
SUV_max_	8.35	2.74 to 25.45	<0.001 *

RBC, red blood cells; WBC, white blood cells; AST, aspartate transaminase; ALT, alanine transaminase; eGFR, estimated glomerular filtration rate; LDH, lactate dehydrogenase, AP, alkaline phosphatase; PSA, prostate-specific antigen; PSMA, prostate-specific membrane antigen; TV, tumor volume; TL, total lesions; SUV, standardized uptake value. * reached significance.

**Table 3:** Multivariate logistic regression analysis.

Variable	Hazard Ratio	95% CI	*p*-Value
Age	8.05	2.15 to 30.09	0.002 *
Hepatic metastases	0.22	0.03 to 1.57	0.132
SUV_max_	7.94	2.25 to 28.05	0.001 *

SUV_max_, maximum standardized uptake value. * reached significance.

## Data Availability

The data are not publicly available because, due to the European regulations regarding data protection, we cannot make data available online or disburse them. However, all data are available for revision on-site.

## References

[1] Rahbar K., Ahmadzadehfar H., Kratochwil C., Haberkorn U., Schafers M., Essler M., Baum R.P., Kulkarni H.R., Schmidt M., Drzezga A. (2017). German Multicenter Study Investigating 177Lu-PSMA-617 Radioligand Therapy in Advanced Prostate Cancer Patients. J. Nucl. Med..

[2] Hofman M.S., Violet J., Hicks R.J., Ferdinandus J., Thang S.P., Akhurst T., Iravani A., Kong G., Ravi Kumar A., Murphy D.G. (2018). [(177)Lu]-PSMA-617 radionuclide treatment in patients with metastatic castration-resistant prostate cancer (LuPSMA trial): A single-centre, single-arm, phase 2 study. Lancet Oncol..

[3] Fendler W.P., Calais J., Eiber M., Flavell R.R., Mishoe A., Feng F.Y., Nguyen H.G., Reiter R.E., Rettig M.B., Okamoto S. (2019). Assessment of 68Ga-PSMA-11 PET Accuracy in Localizing Recurrent Prostate Cancer: A Prospective Single-Arm Clinical Trial. JAMA Oncol..

[4] Giesel F.L., Will L., Lawal I., Lengana T., Kratochwil C., Vorster M., Neels O., Reyneke F., Haberkon U., Kopka K. (2018). Intraindividual Comparison of (18)F-PSMA-1007 and (18)F-DCFPyL PET/CT in the Prospective Evaluation of Patients with Newly Diagnosed Prostate Carcinoma: A Pilot Study. J. Nucl. Med..

[5] Giesel F.L., Knorr K., Spohn F., Will L., Maurer T., Flechsig P., Neels O., Schiller K., Amaral H., Weber W.A. (2019). Detection Efficacy of (18)F-PSMA-1007 PET/CT in 251 Patients with Biochemical Recurrence of Prostate Cancer After Radical Prostatectomy. J. Nucl. Med..

[6] Watabe T., Uemura M., Soeda F., Naka S., Ujike T., Hatano K., Sasaki H., Kamiya T., Shimosegawa E., Kato H. (2021). High detection rate in [(18)F]PSMA-1007 PET: Interim results focusing on biochemical recurrence in prostate cancer patients. Ann. Nucl. Med..

[7] Sawicki L.M., Kirchner J., Buddensieck C., Antke C., Ullrich T., Schimmoller L., Boos J., Schleich C., Schaarschmidt B.M., Buchbender C. (2019). Prospective comparison of whole-body MRI and (68)Ga-PSMA PET/CT for the detection of biochemical recurrence of prostate cancer after radical prostatectomy. Eur. J. Nucl. Med. Mol. Imaging.

[8] Rowe S.P., Macura K.J., Mena E., Blackford A.L., Nadal R., Antonarakis E.S., Eisenberger M., Carducci M., Fan H., Dannals R.F. (2016). PSMA-Based [(18)F]DCFPyL PET/CT Is Superior to Conventional Imaging for Lesion Detection in Patients with Metastatic Prostate Cancer. Mol. Imaging Biol..

[9] Kratochwil C., Giesel F.L., Stefanova M., Benesova M., Bronzel M., Afshar-Oromieh A., Mier W., Eder M., Kopka K., Haberkorn U. (2016). PSMA-Targeted Radionuclide Therapy of Metastatic Castration-Resistant Prostate Cancer with 177Lu-Labeled PSMA-617. J. Nucl. Med..

[10] Baum R.P., Kulkarni H.R., Schuchardt C., Singh A., Wirtz M., Wiessalla S., Schottelius M., Mueller D., Klette I., Wester H.J. (2016). 177Lu-Labeled Prostate-Specific Membrane Antigen Radioligand Therapy of Metastatic Castration-Resistant Prostate Cancer: Safety and Efficacy. J. Nucl. Med..

[11] Hofman M.S., Emmett L., Sandhu S., Iravani A., Joshua A.M., Goh J.C., Pattison D.A., Tan T.H., Kirkwood I.D., Ng S. (2021). [(177)Lu]Lu-PSMA-617 versus cabazitaxel in patients with metastatic castration-resistant prostate cancer (TheraP): A randomised, open-label, phase 2 trial. Lancet.

[12] Rahbar K., Bodei L., Morris M.J. (2019). Is the Vision of Radioligand Therapy for Prostate Cancer Becoming a Reality? An Overview of the Phase III VISION Trial and Its Importance for the Future of Theranostics. J. Nucl. Med..

[13] Manafi-Farid R., Harsini S., Saidi B., Ahmadzadehfar H., Herrmann K., Briganti A., Walz J., Beheshti M. (2021). Factors predicting biochemical response and survival benefits following radioligand therapy with [(177)Lu]Lu-PSMA in metastatic castrate-resistant prostate cancer: A review. Eur. J. Nucl. Med. Mol. Imaging.

[14] Seifert R., Seitzer K., Herrmann K., Kessel K., Schafers M., Kleesiek J., Weckesser M., Boegemann M., Rahbar K. (2020). Analysis of PSMA expression and outcome in patients with advanced Prostate Cancer receiving (177)Lu-PSMA-617 Radioligand Therapy. Theranostics.

[15] Seifert R., Kessel K., Schlack K., Weber M., Herrmann K., Spanke M., Fendler W.P., Hadaschik B., Kleesiek J., Schafers M. (2021). PSMA PET total tumor volume predicts outcome of patients with advanced prostate cancer receiving [(177)Lu]Lu-PSMA-617 radioligand therapy in a bicentric analysis. Eur. J. Nucl. Med. Mol. Imaging.

[16] Gafita A., Calais J., Wang H., Weber M., Rathke H., Esfandiari R., Armstrong W., Kratochwil C., Tauber R., Delpassand E. (2020). Predictive factors and prediction nomograms for LuPSMA radioligand therapy in patients with metastatic castration-resistant prostate cancer: An international multicentre retrospective study. J. Nucl. Med..

[17] Derlin T., Werner R.A., Lafos M., Henkenberens C., von Klot C.A.J., Sommerlath Sohns J.M., Ross T.L., Bengel F.M. (2020). Neuroendocrine Differentiation and Response to PSMA-Targeted Radioligand Therapy in Advanced Metastatic Castration-Resistant Prostate Cancer: A Single-Center Retrospective Study. J. Nucl. Med..

[18] Schmuck S., von Klot C.A., Henkenberens C., Sohns J.M., Christiansen H., Wester H.J., Ross T.L., Bengel F.M., Derlin T. (2017). Initial Experience with Volumetric (68)Ga-PSMA I&T PET/CT for Assessment of Whole-Body Tumor Burden as a Quantitative Imaging Biomarker in Patients with Prostate Cancer. J. Nucl. Med..

[19] Fendler W.P., Kratochwil C., Ahmadzadehfar H., Rahbar K., Baum R.P., Schmidt M., Pfestroff A., Lutzen U., Prasad V., Heinzel A. (2016). [177Lu-PSMA-617 therapy, dosimetry and follow-up in patients with metastatic castration-resistant prostate cancer]. Nuklearmedizin.

[20] Scher H.I., Halabi S., Tannock I., Morris M., Sternberg C.N., Carducci M.A., Eisenberger M.A., Higano C., Bubley G.J., Dreicer R. (2008). Design and end points of clinical trials for patients with progressive prostate cancer and castrate levels of testosterone: Recommendations of the Prostate Cancer Clinical Trials Working Group. J. Clin. Oncol..

[21] Hachamovitch R., Di Carli M.F. (2008). Methods and limitations of assessing new noninvasive tests: Part II: Outcomes-based validation and reliability assessment of noninvasive testing. Circulation.

[22] Kim Y.J., Kim Y.I. (2018). Therapeutic Responses and Survival Effects of 177Lu-PSMA-617 Radioligand Therapy in Metastatic Castrate-Resistant Prostate Cancer: A Meta-analysis. Clin. Nucl. Med..

[23] Rathke H., Holland-Letz T., Mier W., Flechsig P., Mavriopoulou E., Rohrich M., Kopka K., Hohenfellner M., Giesel F.L., Haberkorn U. (2020). Response Prediction of (177)Lu-PSMA-617 Radioligand Therapy Using Prostate-Specific Antigen, Chromogranin A, and Lactate Dehydrogenase. J. Nucl. Med..

[24] Derlin T., Sommerlath Sohns J.M., Schmuck S., Henkenberens C., von Klot C.A.J., Ross T.L., Bengel F.M. (2020). Influence of short-term dexamethasone on the efficacy of (177) Lu-PSMA-617 in patients with metastatic castration-resistant prostate cancer. Prostate.

[25] Gadot M., Davidson T., Aharon M., Atenafu E.G., Malki A., Levartovsky M., Saad A., Domachevsky L., Berger R., Leibowitz R. (2020). Clinical Variables Associated with PSA Response to Lutetium-177-PSMA ([177Lu]-PSMA-617) Radionuclide Treatment in Men with Metastatic Castration-Resistant Prostate Cancer. Cancers.

[26] Yadav M.P., Ballal S., Bal C., Sahoo R.K., Damle N.A., Tripathi M., Seth A. (2020). Efficacy and Safety of 177Lu-PSMA-617 Radioligand Therapy in Metastatic Castration-Resistant Prostate Cancer Patients. Clin. Nucl. Med..

[27] Gafita A., Heck M.M., Rauscher I., Tauber R., Cala L., Franz C., D’Alessandria C., Retz M., Weber W.A., Eiber M. (2020). Early Prostate-Specific Antigen Changes and Clinical Outcome After (177)Lu-PSMA Radionuclide Treatment in Patients with Metastatic Castration-Resistant Prostate Cancer. J. Nucl. Med..

[28] Michalski K., Mix M., Meyer P.T., Ruf J. (2019). Determination of whole-body tumour burden on [68Ga]PSMA-11 PET/CT for response assessment of [177Lu]PSMA-617 radioligand therapy: A retrospective analysis of serum PSA level and imaging derived parameters before and after two cycles of therapy. Nuklearmedizin.

[29] Michalski K., Ruf J., Goetz C., Seitz A.K., Buck A.K., Lapa C., Hartrampf P.E. (2021). Prognostic implications of dual tracer PET/CT: PSMA ligand and [(18)F]FDG PET/CT in patients undergoing [(177)Lu]PSMA radioligand therapy. Eur. J. Nucl. Med. Mol. Imaging.

[30] Perez P.M., Hope T.A., Behr S.C., van Zante A., Small E.J., Flavell R.R. (2019). Intertumoral Heterogeneity of 18F-FDG and 68Ga-PSMA Uptake in Prostate Cancer Pulmonary Metastases. Clin. Nucl. Med..

[31] Chan D.L., Pavlakis N., Schembri G.P., Bernard E.J., Hsiao E., Hayes A., Barnes T., Diakos C., Khasraw M., Samra J. (2017). Dual Somatostatin Receptor/FDG PET/CT Imaging in Metastatic Neuroendocrine Tumours: Proposal for a Novel Grading Scheme with Prognostic Significance. Theranostics.

[32] Kratochwil C., Fendler W.P., Eiber M., Baum R., Bozkurt M.F., Czernin J., Delgado Bolton R.C., Ezziddin S., Forrer F., Hicks R.J. (2019). EANM procedure guidelines for radionuclide therapy with (177)Lu-labelled PSMA-ligands ((177)Lu-PSMA-RLT). Eur. J. Nucl. Med. Mol. Imaging.

[33] Wang G., Zhao D., Spring D.J., DePinho R.A. (2018). Genetics and biology of prostate cancer. Genes Dev..

[34] Kamel M.H., Khalil M.I., Alobuia W.M., Su J., Davis R. (2018). Incidence of metastasis and prostate-specific antigen levels at diagnosis in Gleason 3+4 versus 4+3 prostate cancer. Urol. Ann..

[35] Han S., Woo S., Kim Y.I., Lee J.L., Wibmer A.G., Schoder H., Ryu J.S., Vargas H.A. (2021). Concordance between Response Assessment Using Prostate-Specific Membrane Antigen PET and Serum Prostate-Specific Antigen Levels after Systemic Treatment in Patients with Metastatic Castration Resistant Prostate Cancer: A Systematic Review and Meta-Analysis. Diagnostics.

[36] Adams M.C., Turkington T.G., Wilson J.M., Wong T.Z. (2010). A systematic review of the factors affecting accuracy of SUV measurements. AJR Am. J. Roentgenol..

[37] Lawhn-Heath C., Salavati A., Behr S.C., Rowe S.P., Calais J., Fendler W.P., Eiber M., Emmett L., Hofman M.S., Hope T.A. (2021). Prostate-specific Membrane Antigen PET in Prostate Cancer. Radiology.

[38] Ceci F., Oprea-Lager D.E., Emmett L., Adam J.A., Bomanji J., Czernin J., Eiber M., Haberkorn U., Hofman M.S., Hope T.A. (2021). E-PSMA: The EANM standardized reporting guidelines v1.0 for PSMA-PET. Eur. J. Nucl. Med. Mol. Imaging.

[39] Eiber M., Herrmann K., Calais J., Hadaschik B., Giesel F.L., Hartenbach M., Hope T., Reiter R., Maurer T., Weber W.A. (2018). Prostate Cancer Molecular Imaging Standardized Evaluation (PROMISE): Proposed miTNM Classification for the Interpretation of PSMA-Ligand PET/CT. J. Nucl. Med..

[40] Rowe S.P., Pienta K.J., Pomper M.G., Gorin M.A. (2018). PSMA-RADS Version 1.0: A Step Towards Standardizing the Interpretation and Reporting of PSMA-targeted PET Imaging Studies. Eur. Urol..

